# Cytomegalovirus (CMV) seroprevalence among women at childbearing age, maternal and congenital CMV infection: policy implications of a descriptive, retrospective, community-based study

**DOI:** 10.1186/s13584-023-00566-9

**Published:** 2023-04-25

**Authors:** Assaf Ben Shoham, Yechiel Schlesinger, Ian Miskin, Ziva Kalderon, Rachel Michaelson-Cohen, Yonit Wiener-Well

**Affiliations:** 1 Clalit Health Services, Yehuda Burla 26/28, 9371426 Jerusalem, Israel; 2 Wilf Children’s Hospital, Shaare Zedek Medical Center, Faculty of Medicine, Hebrew University of Jerusalem, Jerusalem, Israel; 3 Clalit Health Services, Faculty of Medicine, Hebrew University of Jerusalem, Jerusalem, Israel; 4 Shaare Zedek Medical Center, Faculty of Medicine, Hebrew University of Jerusalem, Jerusalem, Israel

**Keywords:** Cytomegalovirus, Seroprevalence, Primary infection, Non-primary infection, Congenital cytomegalovirus infection

## Abstract

**Background:**

Maternal CMV infection during pregnancy, either primary or non-primary, may be associated with fetal infection and long-term sequelae. While guidelines recommend against it, screening for CMV in pregnant women is a prevalent clinical practice in Israel. Our aim is to provide updated, local, clinically relevant, epidemiological information about CMV seroprevalence among women at childbearing age, the incidence of maternal CMV infection during pregnancy and the prevalence of congenital CMV (cCMV), as well as to provide information about the yield of CMV serology testing.

**Methods:**

We performed a descriptive, retrospective study of women at childbearing age who were members of Clalit Health Services in the district of Jerusalem and had at least one gestation during the study period (2013–2019). We utilized serial serology tests to determine CMV serostatus at baseline and at pre/periconception and identified temporal changes in CMV serostatus. We then conducted a sub-sample analysis integrating inpatient data on newborns of women who gave birth in a single large medical center. cCMV was defined as either positive urine CMV-PCR test in a sample collected during the first 3 weeks of life, neonatal diagnosis of cCMV in the medical records, or prescription of valganciclovir during the neonatal period.

**Results:**

The study population Included 45,634 women with 84,110 associated gestational events. Initial CMV serostatus was positive in 89% women, with variation across different ethno-socioeconomic subgroups. Based on consecutive serology tests, the detected incidence rate of CMV infection was 2/1000 women follow-up years, among initially seropositive women, and 80/1000 women follow-up years, among initially seronegative women. CMV infection in pregnancy was identified among 0.2% of women who were seropositive at pre/periconception and among 10% of women who were seronegative. In a subsample, which included 31,191 associated gestational events, we identified 54 newborns with cCMV (1.9/1000 live births). The prevalence of cCMV among newborns of women who were seropositive at pre/periconception was lower than among newborns of women who were seronegative (2.1 vs. 7.1/1000). Frequent serology tests among women who were seronegative at pre/periconception detected most primary CMV infections in pregnancy that resulted in cCMV (21/24). However, among women who were seropositive, serology tests prior to birth detected none of the non-primary infections that resulted in cCMV (0/30).

**Conclusions:**

In this retrospective community-based study among women of childbearing age characterized by multiparity and high seroprevalence of CMV, we find that consecutive CMV serology testing enabled to detect most primary CMV infections in pregnancy that led to cCMV in newborns but failed to detect non-primary CMV infections in pregnancy. Conducting CMV serology tests among seropositive women, despite guidelines' recommendations, has no clinical value, while it is costly and introduces further uncertainties and distress. We thus recommend against routine CMV serology testing among women who were seropositive in a prior serology test. We recommend CMV serology testing prior to pregnancy only among women known to be seronegative or women whose serology status is unknown.

**Supplementary Information:**

The online version contains supplementary material available at 10.1186/s13584-023-00566-9.

## Background

Congenital Cytomegalovirus (cCMV) is the most common congenital infection in developed countries. It is estimated that 0.7% of all newborns in the US are born with cCMV [1–3], and similar rates were reported in Israel [4, 5]. Although nearly 90% of congenitally infected newborns are asymptomatic at birth, cCMV infection may lead to severe long-term sequelae and it is a primary cause of neuro-developmental disabilities. It is estimated that 15%-20% of congenitally infected newborns are born with or develop impairments such as sensorineural hearing loss, vision loss, cerebral palsy, or cognitive impairment. [1, 6].

Maternal CMV infection during pregnancy is either primary or non-primary. Primary infection is defined when a pregnant woman is infected for the first time around conception or during pregnancy, whereas non-primary infection is defined when either reactivation of an existing latent endogenous virus occurs (recurrence) or an infection with a different strain of CMV (reinfection) is diagnosed during pregnancy in a woman who was seropositive prior to conception [7, 8]. Practically, pregnant women are considered to have primary infection when anti-CMV IgG seroconversion occurs during pregnancy. It is estimated that 50–70% of US women under age 45 are CMV seropositive [3, 9]. In Israel, seropositivity among pregnant women is estimated at 80–85% [4, 5, 10].

Both primary and non-primary infections are associated with fetal infection. The risk of vertical transmission after primary infection is estimated at 30–40% while the risk of transmission in non-primary infection during pregnancy was historically estimated at 1% [1]. However, recent studies report this rate to be as high as 20% [11]. The risk of transmission is associated with gestational age during maternal infection, estimated at less than 5% when maternal CMV infection occurs at periconception, increasing gradually throughout the first, second and third trimester, by some estimates, to 35%, 42% and 59%, respectively [12–15].

Fetal infection is confirmed during pregnancy by PCR test for CMV DNA or by viral culture of the amniotic fluid. In the neonatal period, a definite diagnosis of cCMV requires direct viral detection in saliva, urine, or blood samples during the first 3 weeks of life [16, 17]. There is no recommendation for universal screening for cCMV in the newborn and, at present, testing is based largely on clinical suspicion or following serological evidence of maternal infection [4, 16].

Screening pregnant women for CMV serology in not recommended by the CDC, and the Israeli ministry of health also advise against routine CMV serology testing during pregnancy [3]. Despite this guidance, numerous Israeli women are being tested repeatedly before and during pregnancy.

Our aim is to provide local, population-based information about CMV seroprevalence among pregnant women, the incidence of primary and non-primary maternal CMV infection during pregnancy and the prevalence of cCMV. The yield of CMV serology during pregnancy is examined, elucidating the basis for a policy recommending against serology based maternal screening.

## Methods

### Study population

This is a descriptive, retrospective, community-based study based on data about women of childbearing age and newborns from outpatient and inpatient medical records. The data was retrieved from records in Jerusalem district of Clalit Health Services (CHS) which provides healthcare services to over 500,000 insurees. A sub-sample includes data on newborns of women who gave birth in Shaare-Zedek Medical Center (SZMC), the largest delivery center in Jerusalem, with a mean annual volume of 20,000 births, comprising approximately 16% of all deliveries in Israel.

We included women of childbearing age (18–44), insured by CHS and registered in the district of Jerusalem, who had at least one gestational outcome during 2013–2019 (the study period). A gestational outcome (a case) is defined as either a registered live birth or code-identified in-hospital procedure documented in the electronic medical record (EMR) specified as dilation and curettage (D&C) or induced abortion (surgical or pharmacological, including intra-amniotic injection for late abortion). The number of gestational outcomes (cases) exceeds the number of women in the sample, since many women in the study population had multiple gestational outcomes during the study period.

### Data and measures

For each case, demographic and clinical data were extracted from the EMR, including age and ethnicity, pregnancy associated procedures (abortion, D&C, termination of pregnancy or delivery), and all CMV lab serology tests (IgM, IgG, IgG-avidity) during 2010–2020, overlapping the study period. The overlap facilitated interpretation and classification of the serologic status (serostatus) at baseline, before, during and after each gestational event, when the data permitted.

Since the detection of CMV infection may depends on the frequency of serology testing, we calculated the mean number of tests during 2010–2020 and the frequency of testing (the number of CMV tests divided by the number of years between first and last CMV tests).

For each case (gestation), we considered longitudinal data of CMV serology test results to define the serostatus at baseline and prior to gestation (i.e., at pre/periconception). We further defined CMV infection in pregnancy, relevant to a specific gestation, if seroconversion or serologic evidence of non-primary infection were found during periconception or during pregnancy (within 10 months prior to the date of the documented gestational outcome).

CHS' laboratories implement a reflexive protocol for confirming CMV-IgM serology test results when a sample is taken from a woman of childbearing age [18]. An analyzer is used first to screen for positive CMV-IgM, and if the result is borderline or positive, a confirmation test is performed with CMV-IgM-VIDAS analyzer. Further, when both validated CMV-IgM and CMV-IgG are positive, CMV-IgG avidity test is performed whenever the patient is a woman at childbearing age with a positive IgG test result for the first time. High avidity suggests CMV infection that occurred at least 3 months before the sampling date whereas low avidity indicates recent infection. The test results are reported in text (*Negative*, *Borderline* or *Positive*), or inferred from the numeric result according to laboratory defined cutoff values (Additional file 1: Tables S1–S4 supplement A).

Based on validated CMV-IgM, IgG and IgG avidity test results, we defined the CMV point serostatus associated with each serology test array (Additional file 1: Table S5 supplement A).

Next, we examined consecutive CMV serology tests to identify changes in CMV point serostatus. Some changes were easily classified (e.g., *Uninfected* to *Past infection*, indicating a primary infection during the time-period between the two consecutive tests), whereas other changes in CMV point serostatus called for inspection of a broader history of consecutive test results (e.g., *Past infection* to *Tail/Persistent IgM/Non-primary*). For those changes, which were not straightforward, four authors (YWW, IM, YS, AB) independently inspected the history of CMV serology tests to reach a decision about classification. The final classification included the categories *no change*, *seroconversion* (due to primary infection) and *non-primary infection*.cCMV was defined for the subsample if one or more of the following applied: positive urine CMV-PCR test in a sample collected during the first three weeks of life, neonatal diagnosis of cCMV in the medical records (either at discharge from SZMC or in ambulatory follow up in CHS) or prescription of valganciclovir during the neonatal period.

We extracted descriptive and comparative statistics using relevant appropriate statistical tests. Analysis was performed using SPSS software, version 25 (SPSS, Chicago IL, USA).

The study was approved by both SZMC and CHS ethical review boards prior to data collection.

## Results

### Study population and CMV infection during 2010–2020

We identified 45,634 women with at least one gestational outcome documented during the study period. The entire cohort collectively had more than 160,000 CMV serology tests during 2010–2020 and the majority (95%) of women in the study population had at least one CMV serology blood test. Table 1 shows demographic and gestational characteristics of the study population, initial CMV serostatus and CMV infection during 2010–2020, for the three major population subgroups residing in the district.

**Table 1:** The study population, initial CMV serologic status and CMV infection during 2010–2020

	Ultra-orthodox Jews	Arab	General	All	*P* value
Number of women (row %)	12,589 (28%)	16,333 (36%)	16,712 (37%)	45,634	
NSI exemption	8%	NA	6%	7%	< 0.001
Age at first gestational event during 2013–2019 (mean)	28.1	26.3	29.9	28.1	< 0.001
Pregnancies during 2013–2019 (mean)	2.2	1.7	1.8	1.9	< 0.001
1 pregnancy	30%	47%	42%	41%	< 0.001
> 3 pregnancies	11%	3%	4%	6%	< 0.001
Live births (mean)	2.0	1.5	1.6	1.7	< 0.001
Abortion or termination of pregnancies (mean)	0.18	0.19	0.25	0.21	< 0.001
CMV lab tests available	96%	96%	93%	95%	< 0.001
Seropositive initial CMV serostatus	86%	98%	83%	89%	< 0.001
CMV infection during 2010–2020	6.5%	0.8%	6.6%	4.4%	< 0.001

NSI = National Security Institute exemption indicator is associated with eligibility for discount or waiver on select services' fees and may serve as a proxy for low socio-economic status as well as utilization and exhaustion of rights. This is not a good indicator for socio-economic status in Arab population for various reasons

Seropositivity was highest among Arab Israeli women (98%) and lowest among women of the general population (83%). The rate of CMV infection, defined as either seroconversion or a non-primary infection, was lower among Arab Israeli women (0.8%). Table 2 shows descriptive statistics of CMV serology testing and CMV infection by initial CMV serostatus.

**Table 2:** CMV serology tests and CMV infection during 2010–2020, by initial CMV serostatus

	Initial CMV serostatus		*p* value
	Seropositive	Seronegative	
Number of women (row %)	38,622 (89%)	4635 (11%)	
CMV serology tests (mean ± SD)	3.2 ± 2.0	6.2 ± 4.0	< 0.001
Frequency of CMV serology tests (mean ± SD)*	1.1 ± 2.3	2.1 ± 2.9	< 0.001
CMV infection (%)	292 (0.8%)	1,633 (35.2%)	< 0.001

*Frequency of CMV serology tests is defined as the number of CMV tests divided by the number of years between first and last CMV tests. When only a single test exists, test frequency is set to zero

Women who were initially seropositive had fewer CMV tests during 2010–2020 compared to women who were initially seronegative. This finding was consistent within population subgroups, with more CMV tests among ultra-orthodox women (6.5 and 3.9 tests during the study period, among initially seronegative and seropositive women, respectively), and fewer among Arab women (3.1 and 2.7 tests during the study period, among women who were initially seronegative and seropositive, respectively, not shown).

During 2010–2020, CMV infection was identified in 292 (0.8%) of the seropositive women and in 1633 (35.2%) of the seronegative women. Thus, retrospectively, based on consecutive serology testing alone, most identified CMV infections were primary (1633/1925, 84.8%) and the minority non-primary (292/1,925, 15.2%). The risk of CMV infection was lower among initially seropositive women (0.2 infections per 100 women follow-up years vs. 8 among initially seronegative women, OR 0.023, *p* value < 0.001), as well as among women at older age brackets (OR 0.78 among 25–34, OR 0.65 among 35–44, *p* value < 0.001, relative to women who were 18–24; Additional file 1: Tables S6 and S7 supplement B).

### CMV infection in pregnancy, based on longitudinal CMV serology tests

For each pregnancy, we defined CMV serostatus at pre/periconception, according to serology tests performed at least 10 months prior to that gestational event (e.g., date of gestational outcome). Seropositivity at pre/periconception was observed in 90% of the pregnancies (Table 3). The incidence rate of CMV infection during pregnancy, based on consecutive serology tests alone, was 12/1000 pregnancies: non-primary CMV infection in pregnancy was found among 0.2% of seropositive women, while primary infection was found among 10.3% of the seronegative women.

**Table 3:** CMV infection during pregnancy and gestational outcomes, by prepericonceptional serostatus

	Pre/periconceptional serostatus		All	*P* value
	Seropositive	Seronegative		
	76,075 (90%)	8035 (10%)	84,110	
Live birth	90.2%	90.8%	90.30%	0.113
Abortion/TOP	9.8%	9.2%	9.70%	
*By CMV infection*				
CMV infection	0.2%	10.3%	1.2%	< 0.001
Live birth	90.8%	91.6%	91.5%	0.768
Abortion/TOP	9.2%	8.4%	8.5%	
No CMV infection	99.8%	89.7%	98.8%	0.209
Live birth	90.2%	90.7%	90.3%	
Abortion/TOP	9.8%	9.3%	9.7%	

*TOP* termination of pregnancy

Termination of pregnancy (TOP) and spontaneous abortions occurred in 9.7% of the pregnancies with no significant difference between seropositive and seronegative women, or among women detected with CMV infection in pregnancy comparing to those not infected (Table 3). Among seronegative women in the ultra-orthodox population subgroup, CMV infection in pregnancy was associated with lower percentage of abortive gestational outcome (5% vs 8.1%, *p* value < 0.04; none of the comparisons among seropositive women were statistically significant due to small number of women whose consecutive serology testing indicated CMV infection during pregnancy in this group; Additional file 1: Table S8 supplement B).

### Subsample analysis with newborns' data from SZMC

We analyzed 31,191 gestational events associated with 15,025 women who gave birth or underwent TOP in SZMC. This subsample represents 33% of all women in the cohort and includes more ultra-orthodox women (44% vs. 28%, *p* < 0.001) and less Arab women (22% vs. 36%, *p* < 0.001) than the entire study population (Additional file 1: Table S9 supplement C).

**Fig. 1:**
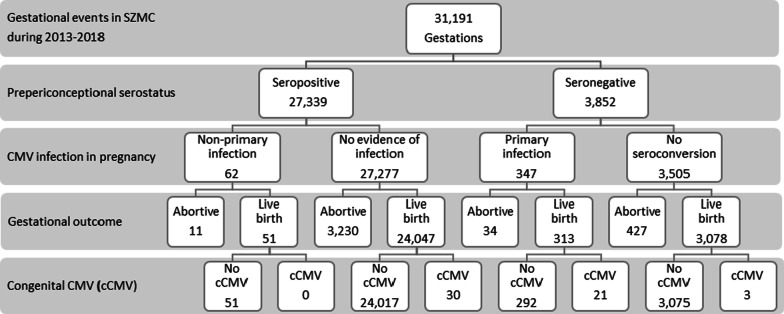
Pre/periconceptional serostatus, detected CMV in pregnancy, outcome of pregnancy and identified cCMV, subsample analysis

Integrating maternal and newborn data, we identified 54 newborns who had cCMV (1.9/1000 live births), of which 30 (56%) were born to seropositive women and 24 (44%) were born to seronegative women (Fig. 1). The prevalence of cCMV among newborns of seropositive women was lower than among newborns of seronegative women (1.2/1000 vs. 7.1/1000 live births, respectively, *p* value < 0.001).

In a multivariable regression analysis for factors associated with cCMV infection, seropositivity before pregnancy was found to be highly protective (OR 0.001, *p* value < 0.001). Being born to mid-range age women [25–34] relative to younger [18–24] or older (35–44) women was also protective. In comparison to other population subgroups, newborns of Arab women were at higher risk for cCMV (OR 2.4 and 398, among seropositive and seronegative, respectively; Additional file 1: Table S10 supplement C).

## Discussion

### Main findings

In this retrospective, community-based study, among childbearing age women in the district of Jerusalem, we find high prevalence of CMV seropositivity with significant differences between its three large ethno-socioeconomic population subgroups.

CMV seroprevalence in the study population is higher than the prevalence described in other developed countries [1–3, 9] and higher than previously reported in Israel [4, 5]. High seroprevalence in the study population may be associated with low socioeconomic status and overcrowding at home, characteristic to Jerusalem district's population. This finding, as well as the differences between ethno-socioeconomic subgroups, is consistent with studies which have previously shown that seroprevalence and infection rates were significantly higher in lower compared to middle or upper household income groups and that there are considerable ethnic and racial disparities in CMV incidence [1–3]. These findings attest to the local epidemiology of CMV. Knowledge of CMV seroprevalence in a specific population is an important input in devising cost-effective strategies for detecting CMV in pregnancy and cCMV.

Utilizing longitudinal serology data alone, we detected CMV infection in pregnancy among 0.2% of pre/periconceptional seropositive women and among 10% of pre/periconceptional seronegative women. That is, 15% of detected CMV infections in pregnancy were non-primary. This finding is inconsistent with data from prospective studies estimating that in developed countries, where seroprevalence is lower than in our study population, 50–75% of CMV infections in pregnancy are due to non-primary infections [19–22]. Moreover, in the subsample analysis, available longitudinal serology data did not enable to detect any of the non-primary CMV infections in pregnancy among mothers to newborns who were diagnosed with cCMV.

There are still many questions about the mechanism of non-primary CMV infection [8, 23] and further uncertainties about the mechanism of vertical transmission [24]. While estimates of the vertical transmission rate following primary infections range from 14 to 52%, the literature reports lower estimates of the vertical transmission rate following non-primary infection, ranging from 1 to 20% [9, 11, 13, 23, 25]. Applying the latter estimate range to our subsample data, one would expect 150 to 3,000 additional cases of non-primary CMV infection, which were undetected by consecutive serology tests as performed in routine community follow-up.

Less and infrequent serology testing among seropositive women may have contributed to the failure to detect non-primary CMV infections (Table 2). However, identifying non-primary infection is difficult and lacking in sensitivity and specificity even with repeated serology tests. Indeed, the CDC and the informal international cCMV recommendations group do not recommend routinely testing pregnant women for CMV and testing is recommended only when a pregnant woman requests the test, a pregnant woman experiences a mononucleosis-like illness, or if fetal anomaly is detected [16, 26, 27]. The Israeli MOH recommends against routine CMV serology testing and restated this recommendation in a circular which also provided guidelines about the interpretation and work up following CMV serology tests which were taken despite this recommendation [4]. Nevertheless, in our study population, of 45,634 women with 84,110 associated gestational events, over 160,000 serology tests were performed during 2010–2020, where 95% of the women had at least one CMV serology test during this period. It seems that the MOH recommendations against routine CMV serology tests are not implemented.

Although multiple serology tests were performed, often prompting intense follow up during pregnancy, the rate of abortions and TOP was not higher among women detected with CMV infection in pregnancy, either primary or non-primary. This finding may reflect the conservative attitude of major subgroups in the study populations regarding decisions on TOP, even when CMV infection in pregnancy had been diagnosed. The only subgroup in which detected CMV infection in pregnancy was associated with an increased rate of abortions and TOP was the general population subgroup, including mostly secular women (not significant; Additional file 1: Table S8 supplement B).

Utilizing newborns' data, we identified 54 newborns with cCMV out of 27,489 live births (2/1000 live births), 56% following non-primary CMV infection in pregnancy and 44% following primary infection. Prevalence of cCMV among newborns of seropositive women was lower than among newborns of seronegative women (1.2/1000 vs. 7.1/1000 live births, respectively, *p* value < 0.001). Estimates of the prevalence of cCMV range from 2 to 20 per 1000 live births, lower in industrialized, low seroprevalence countries and higher in developing and high seroprevalence countries [6, 9, 28]. Prevalence of cCMV in our study is on the lower range of estimates in the literature, most probably because our study is retrospective, whereas most estimates in the literature were derived from prospective studies that included preset protocols for newborn cCMV screening.

Among seronegative pregnant women, frequent serology testing enabled to detect most cCMV cases (21/24), which may have facilitated timely consultation and decisions regarding amniocentesis, imaging, TOP or, recently, treating with high dose valacyclovir [29].

### Strengths

This is a community-based, large scale, retrospective study, in a locality of high medical availability, of a population characterized by multiparity and high prevalence of CMV. The available data about the study population represent real world practices and outcomes. In particular, the vast number of CMV serology tests performed, enabled to follow the natural history of CMV within the study population, i.e., to determine the initial and temporal changes to the serostatus throughout 2010–2020, and elicit estimates of local epidemiological parameters. Moreover, it presents an opportunity to study a proxy for CMV serology screening policy among women at childbearing age.

Our results outline a practice of reflexive screening policy (de-facto), where almost all women at childbearing age undergo CMV serology testing (CHS lab implements a reflexive protocol in performing the test itself), and consecutive serology testing is conducted among women who are screened seronegative. Our findings show that this practice enabled to detect most primary CMV infections in pregnancy that led to cCMV in newborns. The findings also restate that consecutive serology testing fails to detect many (if not the vast majority) of non-primary CMV infections in pregnancy, supporting policy recommendation against routine CMV serology testing, and should thus prompt HMOs' laboratories to decline repeat serology testing for women who were previously seropositive.

### Limitations

This study has several limitations emanating from it being retrospective. First, since serology testing was not performed as part of an established protocol, there is variation in the frequency of serology testing. Evidently, this variation results in some missed primary infections and many non-primary infections. We used serology data only, without clinical data, which might have been revealing about some of the changes in CMV serostatus (although CMV infection may be asymptomatic). In accordance with most previous studies of CMV in pregnancy, we were unable to differentiate between recurrent infection or reactivation.

Second, is the lack of cCMV screening for all newborns. The routine surveillance included newborns whose mothers had serological, clinical, or radiological suspicion of CMV disease, who had stigmata of cCMV (e.g., IUGR, microcephaly, hepatosplenomegaly) or failed the universal newborn hearing screening. Our working definition of cCMV, although broad, relies on proxies (e.g., diagnosis in the EMR, prescription of valganciclovir), since we do not perform screening of all newborns for PCR-CMV we undoubtedly missed asymptomatic or late symptomatic newborns with cCMV. We also lack data about CMV in aborted fetuses. Our results thus underestimate the prevalence of cCMV.

Newborn screening has gained attention in recent years and is still under debate in literature [30–34]. While a survey of the issue is beyond the scope of this paper, our results may contribute to the ongoing debate. A cost-effectiveness study from Japan compared first trimester maternal antibody screening for *primary* CMV with newborn targeted screening (screening for cCMV after failure of newborn hearing screening), and with universal newborn screening for cCMV [35]. Targeted neonatal screening was dominant (cost-saving) compared to the status quo scenario, while universal neonatal screening and maternal screening for primary infection were cost-effective. Since only primary infections were detected in the maternal screening arm and since seroprevalence in Japan is lower (60%) than in Israel [3], the results in our study, about the yield of serology testing in detecting non-primary infections, imply that maternal serology screening tests have worse cost-effectiveness in populations with high seroprevalence. Other studies have examined the feasibility and cost-effectiveness of targeted and universal newborn screening [30–32, 34]. Targeted or universal screening initiatives have been implemented in some places such as the province of Ontario, Canada, and several states in the U.S. This has enabled detection of cCMV in symptomatic as well as asymptomatic neonates. Nonetheless, the benefits and costs of these strategies, both from clinical and ethical perspectives, continue to be the subject of debate [36].

A third limitation is the fact that we lacked data regarding the date of conception, hence we employed a broad definition of CMV infection during pregnancy, i.e.,10 months prior to documented date of gestational outcome (abortive or non-abortive). We thus probably overestimate the number of CMV infections in pregnancy, when some may have occurred before conception.

### Future research

Prospective studies, which include both maternal pre/periconceptional serology testing and newborn CMV screening, may provide better estimates about the prevalence of non-primary cCMV infections. There is a need for better diagnosis modalities for identifying women with non-primary infection during pregnancies, which could facilitate timely consultation regarding the risks and decisions for actions, such as anti CMV medications, amniocentesis and even TOP.

## Conclusion

In this large scale, retrospective, community-based study among women of childbearing age characterized by multiparity and high seroprevalence of CMV, we find that consecutive CMV serology testing enables to detect most primary CMV infections in pregnancy that led to cCMV in newborns but fails to detect non-primary CMV infections in pregnancy and to provide timely information about the risk of cCMV among pre/periconceptional seropositive women, even when multiple serology tests are performed. Since CMV infection may be asymptomatic, we lack an efficient way to identify these women, who are associated with most of the infections leading to cCMV in a population with high seroprevalence.

Conducting CMV serology tests among seropositive women, despite guidelines' recommendations, has no clinical value, while it is costly and introduces further uncertainties and distress. We thus recommend against routine CMV serology testing among women who were seropositive in a prior serology test.

We recommend CMV serology testing prior to pregnancy among women known to be seronegative or women whose serology status is unknown. Women found to be seronegative in the test should continue serology follow-up, while women found to be seropositive should not retest routinely during pregnancy or in future pregnancies.

## Supplementary Information

Additional file 1: Supplement A. Definitions of CMV serostatus and CMV infection. Table S1: Cut-off values for classification of CMV IgM, IgG serology test results during 2010-2020. Table S2: Cut-off values for classification of CMV IgG-avidity serology test results during 2010-2020. Table S3: Cross-table of Liaison XL/Architect CMV-IgM and CMV-IgM-VIDAS test results, whenever both tests are available. Table S4: Definition of validated CMV-IgM. Table S5: Definitions of point CMV serostatus according to serology test array. Supplement B. CMV infection based on consecutive serology tests during 2010-2020. Table S6: Incidence of CMV infection during 2010-2020, by initial serostatus. Table S7: Cox regression: CMV infection during 2010-2020. Table S8: Abortion or termination of pregnancyby CMV infection during pregnancy, by subpopulation. Supplement C. Subsample analysis. Table S9: Comparison of women who gave birth in SZMC with those who gave birth in other hospitals; demographics, serostatus and outcomes. Table S10: cCMV infection within live births among the subsample: logistic regression, multiple models.

## Data Availability

The dataset used in the current study is not publicly available due to data sharing policy of both institutions but is available from the corresponding author on reasonable request.

## Abbreviations

CMV: Cytomegalovirus
cCMV: Congenital CMV
SZMC: Shaare Zedek medical center
D&C: Dilation and curettage
TOP: Termination of pregnancy
MOH: Ministry of health
OR: Odds ratio
SD: Standard deviation
IUGR: Intrauterine growth restriction
