# Correction: Cytomegalovirus (CMV) seroprevalence among women at childbearing age, maternal and congenital CMV infection: policy implications of a descriptive, retrospective, community-based study

**DOI:** 10.1186/s13584-023-00571-y

**Published:** 2023-05-25

**Authors:** Assaf Ben Shoham, Yechiel Schlesinger, Ian Miskin, Ziva Kalderon, Rachel Michaelson-Cohen, Yonit Wiener-Well

**Affiliations:** 1 Clalit Health Services, Yehuda Burla 26/28, 9371426 Jerusalem, Israel; 2 Wilf Children’s Hospital, Shaare Zedek Medical Center, Faculty of Medicine, Hebrew University of Jerusalem, Jerusalem, Israel; 3 Clalit Health Services, Faculty of Medicine, Hebrew University of Jerusalem, Jerusalem, Israel; 4 Shaare Zedek Medical Center, Faculty of Medicine, Hebrew University of Jerusalem, Jerusalem, Israel

**Correction: Israel Journal of Health Policy Research (2023) 12:16** 10.1186/s13584-023-00566-9

The original publication of this article contained an incorrect author name. The incorrect and correct information is listed in this correction article, the original article has been updated.

Incorrect

Michelson-Cohen

Correct

Michaelson-Cohen

