# Supplementary material for: Cytomegalovirus (CMV) seroprevalence among women at childbearing age, maternal and congenital CMV infection: policy implications of a descriptive, retrospective, community-based study
Source: Isr J Health Policy Res. 2023 Apr 25;12:16. doi: 10.1186/s13584-023-00566-9 (PMC10131385; doi:10.1186/s13584-023-00566-9)
Supplement: Supplementary file 1 — Additional file 1: Supplement A. Definitions of CMV serostatus and CMV infection. Table S1: Cut-off values for classification of CMV IgM, IgG serology test results during 2010-2020. Table S2: Cut-off values for classification of CMV IgG-avidity serology test results during 2010-2020. Table S3: Cross-table of Liaison XL/Architect CMV-IgM and CMV-IgM-VIDAS test results, whenever both tests are available. Table S4: Definition of validated CMV-IgM. Table S5: Definitions of point CMV serostatus according to serology test array. Supplement B. CMV infection based on consecutive serology tests during 2010-2020. Table S6: Incidence of CMV infection during 2010-2020, by initial serostatus. Table S7: Cox regression: CMV infection during 2010-2020. Table S8: Abortion or termination of pregnancyby CMV infection during pregnancy, by subpopulation. Supplement C. Subsample analysis. Table S9: Comparison of women who gave birth in SZMC with those who gave birth in other hospitals; demographics, serostatus and outcomes. Table S10: cCMV infection within live births among the subsample: logistic regression, multiple models. [file 13584_2023_566_MOESM1_ESM.docx]

**Supplements**

Supplement A: Definitions of CMV serostatus and CMV infection

Until October 2017, CMV serology lab tests were performed with Liaison XL analyzer. Since October 2017, these tests have been conducted with Architect analyzer (Abbot) with appropriate testing kits. CMV-IgM-VIDAS test was performed on BioMérieux analyzer, employing Enzyme Linked Fluorescent Assay technology.

Table S1: Cut-off values for classification of CMV IgM, IgG serology test results during 2010-2020

| CMV IgG | CMV IgM |  |
| --- | --- | --- |
| <0.4 Negative  0.4-0.6 Borderline  >0.6 Positive | <15 Negative  15-30 Borderline  >30 Positive | Prior to April 6, 2012  Liaison XL |
| <12 Negative  12-14 Borderline  >14 Positive | <18 Negative  18-22 Borderline  >22 Positive | From April 6, 2012  until October 10, 2017  Liaison XL |
| <6 Negative  ≥6 Positive | <0.85 Negative  0.85-1 Borderline  >1 Positive | After October 10, 2017  Architect |

Table S2: Cut-off values for classification of CMV IgG-avidity serology test results during 2010-2020

| CMV IgG Avidity |  |
| --- | --- |
| <0.2 Low  0.2-0.8 Moderate  >0.8 High | Prior to December 2, 2011 (VIDAS) |
| <0.2 Low  0.2-0.3 Moderate  >0.3 High | From December 2, 2011 until July 30, 2019 (Liaison) |
| <0.4 Low  0.4-0.65 Moderate  >0.65 High | After July 30, 2019 (VIDAS) |

Test results conducted in the different analyzers is documented either in text or numerically, with or without accompanying textual interpretation (e.g., < X.Y, "< X.Y *Negative*", X.YZ, *"There are antibodies"*, *"Positive*", *"Borderline"*).

Table S3: Cross-table of Liaison XL/Architect CMV-IgM and CMV-IgM-VIDAS test results, whenever both tests are available

|  | CMV-IgM-Liason XL/Architect | | | |
| --- | --- | --- | --- | --- |
| CVM-IgM-VIDAS | Negative | Borderline | Positive | Total |
| Negative | 1,909 | 1,929 | 1,629 | 5,467 |
|  | 35% | 35% | 30% | 100% |
|  | 91% | 68% | 33% | 55% |
| Borderline | 137 | 620 | 594 | 1,351 |
|  | 10% | 46% | 44% | 100% |
|  | 7% | 22% | 12% | 14% |
| Positive | 53 | 292 | 2,722 | 3,067 |
|  | 2% | 10% | 89% | 100% |
|  | 3% | 10% | 55% | 31% |
| Total | 2,099 | 2,841 | 4,945 | 9,885 |
|  | 21% | 29% | 50% | 100% |
|  | 100% | 100% | 100% | 100% |

Coarsening of the classification, by consolidating 'Borderline' and 'Positive' results, only 54.3% (4,228 out of 7,786) of the Borderline/Positive Liaison XL/Architect CMV-IgM tests are also Borderline/Positive in CMV-IgM-VIDAS. Clearly, these results merit the need for the validation process.

We employed a broad definition of validated CMV-IgM test based on both Liaison XL/Architect and VIDAS tests.

Table S4: Definition of validated CMV-IgM

| Architect CMV-IgM | CMV-IgM-VIDAS | validated CMV-IgM |
| --- | --- | --- |
| Negative | NA | Negative |
| Borderline/Positive | Negative | Negative |
|  | Borderline | Borderline |
|  | Positive | Positive |

Table S5: Definitions of point CMV serostatus according to serology test array

| Validated  CMV IgM* | CMV IgG | CMV IgG avidity | Point CMV serology status |
| --- | --- | --- | --- |
| Negative | Negative | NA | Uninfected |
|  | Positive | NA / high | Past infection |
| Borderline/  Positive | Negative | NA | False Positive/Early primary |
|  | Positive | Low | Primary infection |
|  |  | NA/  Borderline/High | Tail of primary/Persistent IgM/Non-primary infection |

Supplement B: CMV infection based on consecutive serology tests during 2010-2020

Table S6: Incidence of CMV infection during 2010-2020, by initial serostatus

| Initial CMV point serology status | Follow-up period (months) | CMV infections (number) | Infection per 100 women follow-up years |
| --- | --- | --- | --- |
| Seronegative | 245,599 | 1,633 | 8 |
| Seropositive | 1,722,855 | 292 | 0.2 |
| Total | 1,968,454 | 1,925 | 1.2 |

p value < 0.001 comparing seronegative and seropositive women

Table S7: Cox regression: CMV infection during 2010-2020*

|  | B | Exp(B) | p-value |
| --- | --- | --- | --- |
| Subpopulation |  |  |  |
| Arab vs. Ultra-orthodox | -0.598 | 0.550 | <0.001 |
| General vs. Ultra-orthodox | 0.059 | 1.061 | 0.225 |
| Maternal age |  |  |  |
| 25-34 vs 18-24 | -0.245 | 0.783 | <0.001 |
| 35-44 vs. 18-24 | -0.433 | 0.649 | <0.001 |
| Initial CMV serostatus |  |  |  |
| Seropositive vs. seronegative | -3.751 | 0.023 | <0.001 |

*Base: age 18-24, seronegative, ultra-orthodox women

Table S8: Abortion or termination of pregnancy (TOP) by CMV infection during pregnancy, by subpopulation

|  | No CMV infection | CMV infection | p-value |
| --- | --- | --- | --- |
| Ultra-orthodox |  |  |  |
| Seronegative | 8.1% | 5.0% | 0.042 |
| Seropositive | 7.9% | 6.4% | 1.000 |
| Arab |  |  |  |
| Seronegative | 8.4% | 7.1% | 1.000 |
| Seropositive | 9.8% | 2.6% | 0.176 |
| General |  |  |  |
| Seronegative | 10.4% | 11.2% | 0.568 |
| Seropositive | 11.6% | 14.9% | 0.442 |

Supplement C: Subsample analysis

Table S9: Comparison of women who gave birth in SZMC with those who gave birth in other hospitals; demographics, serostatus and outcomes

|  | Delivery not at SZMC | Delivery at SZMC | All | p-values |
| --- | --- | --- | --- | --- |
| Number of women (%) | 30,609 (67%) | 15,025 (33%) | 45,634 |  |
| Population subgroups  Ultra-orthodox  Arab  General | 20%  43%  38% | 44%  22%  35% | 28%  36%  37% | <0.001 |
| NSI exemption | 7% | 8% | 7% | <0.001 |
| Mean age at first pregnancy included (years) | 28.0 | 28.3 | 28.1 | <0.001 |
| Mean no. of pregnancies during research period | 1.8 | 2.1 | 1.9 | <0.001 |
| Mean no. of live births during research period | 1.6 | 1.8 | 1.7 | <0.001 |
| Mean no. of abortion or termination of pregnancies | 0.20 | 0.23 | 0.21 | <0.001 |
| At least one CMV lab test available during 2010-2020 | 93% | 98% | 95% | <0.001 |
| Mean number of CMV serology tests | 3.2 | 4.1 | 3.5 | <0.001 |
| Frequency of CMV serology tests | 1.3 | 1.2 | 1.2 | <0.001 |
| CMV initial serology status – seropositive | 90% | 88% | 89% | <0.001 |
| Mean number of CMV serology tests  Seronegative  Seropositive | 5.8  2.9 | 6.8  3.7 | 6.2  3.2 | <0.001 |
| CMV infection during study period | 3.8% | 5.7% | 4.4% | <0.001 |
| CMV infection during F/U period in initially seronegative women | 916(32%) | 717(40.4%) | 1633(35.2%) | <0.001 |
| CMV infection during F/U period in initially seropositive women | 164(0.6%) | 128(1%) | 292(0.8%) | <0.001 |
| Gestational outcome of abortion or TOP | 9.8% | 10.9% | 10.2% |  |
| Abortion or TOP by serology status prior to event  Seronegative  Seropositive | 9.1%  9.9% | 10.8%  10.9% | 9.7%  10.2% |  |
| Seropositive prior to gestation | 90% (out of 31,215 gestations) | 91% (out of 52,921 gestations) | 90% (out of 84,136 gestations) | <0.001 |
| Abortion or TOP | 12.2% | 10.4% | 11.0% | <0.001 |
| Relevant CMV infection  seronegative women  prior to gestation  seropositive women  prior to gestation | 9.0%  0.2% | 9.0%  0.2% | 9.0%  0.2% | P=1  p=0.31 |
| Abortion or TOP by serostatus prior to gestational event  Seronegative  Seropositive | 12.0%  11.9% | 7.4%  8.6% | 9.4%  9.8% | <0.001 |

NSI = National Security Institute exemption indicator is associated with eligibility for discount or waiver on select services' fees and may serve as a proxy for low social-economic status as well as utilization and exhaustion of rights. This is not a good indicator for social-economic status in Arab population for various reasons; TOP = termination of pregnancy

Table S10: cCMV infection within live births among the subsample: logistic regression, multiple models*

|  | Exp(B) | Sig. | Exp(B) | Sig. | Exp(B) | Sig. |
| --- | --- | --- | --- | --- | --- | --- |
| Initial CMV serostatus |  |  |  |  |  |  |
| Seropositive vs. seronegative | 0.001 | <0.001 | 0.000 | <0.001 | 0.001 | <0.001 |
| Maternal age |  |  |  |  |  |  |
| 25-34 vs 18-24 | 0.151 | <0.001 | 0.211 | <0.001 | 0.189 | <0.001 |
| 35-44 vs. 18-24 | 1.617 | <0.001 | 1.602 | 0.001 | 1.452 | 0.015 |
| Subpopulation |  |  |  |  |  |  |
| Arab vs. UO |  |  | 211.6 | <0.001 | 398.5 | <0.001 |
| General vs. UO |  |  | 1.331 | 0.025 | 1.364 | 0.024 |
| Interaction |  |  |  |  |  |  |
| Arab and seropositive |  |  |  |  | 0.006 | 0.000 |
| General and seropositive |  |  |  |  | 0.865 | 0.764 |
| Cox & Snell R Square | 0.72 |  | 0.73 |  | 0.73 |  |
| Nagelkerke R Square | 0.95 |  | 0.97 |  | 0.97 |  |

* Base: 18-24, seronegative, Ultra-Orthodox (UO) women
